# 

**Published:** 1902-05

**Authors:** 


### AMERICAN MEDICAL ASSOCIATION, SECTION ON STOMATOLOGY.

This Association will convene at Saratoga, N. Y., June 10 to 13 of the present year. Upon another page is published the very full programme of the Section on Stomatology. From the reputation of the writers of papers, there is assured an interesting meeting. Saratoga has many advantages as a place for the meeting of scientific bodies. Its quiet beauty, without extraordinary attractions to draw off the members, is not the least of these, for those in the habit of attending such gatherings know full well that interesting meetings are not usually held in places where there is much to enjoy outside of convention work. Saratoga has no lack of these, but not sufficient to draw members away from the severer labor that called them together.

